# Damage to the macula **associated with** LED-derived blue **laser exposure**: A case report

**DOI:** 10.1186/s12886-017-0448-9

**Published:** 2017-04-24

**Authors:** Lingling Liang, Zhihua Cui, Chengwei Lu, Qian Hao, Yajuan Zheng

**Affiliations:** 1 Department of Ophthalmology, The Second Hospital of Jilin University, Changchun, China; 2 Department of Ophthalmology, The First Hospital of Jilin University, Changchun, China

**Keywords:** Optical coherence tomography, Multifocal electroretinography, Led, Macular photo injury

## Abstract

**Background:**

Light emitting diodes laser is emerging as an important source of light replacing conventional lights. It is widely used for illumination in the bar where young people love to go. But not everyone knows about the light damage to the eye especially to the macula. In this article, we report the case of a macular damage induced by LED-derived blue laser in a bar, studied with optical coherence tomography (OCT) to evaluate the retinal lesion and multifocal electroretinography (mfERG) to evaluate functional damage.

**Case presentation:**

Four days after the photo injury to the right eye, the visual acuity was 0.5. Funduscopy revealed a round red lesion in the macula of the right eye. Fluorescein angiography (FA) revealed no leakage. OCT revealed a deficiency in the center of the fovea. MfERG revealed a reduction of the peak value in the right eye compared to the left eye. One month later, although the vision was 1.0 in the right eye, OCT revealed a hyporeflectivity of the ellipsoid zone. MfERG still showed a reduction of the peak value in the right eye compared to the left eye.

**Conclusion:**

We believe that general knowledge about laser injuries to the eye should be realized widely. We also think in cases of macular laser damage, the recovery of vision can not demonstrate the recovery of the function of photoreceptors.

## Background

Light-emitting diode (LED) technology is increasingly replacing conventional light sources. It is widely used for illumination in the bar where young people love to go. But not everyone knows about damage caused by light to the eye especially to the macula.

There are some cases about macular photo injury from laser pointer in the literature. Lim ME [1] reported a case that a 13-year-old boy looked at the mirror reflection of a beam from a green diode laser with average power output of 154mw. Fundus examination and ancillary tests revealed macular thermal injury. Turaka K [2] reported a case about macular light damage and made a mini review. In the article, the author cited a report from American food and drug association that stated the risk of irreversible eye injuries and skin burns from the hand held laser pointers that emit >5 mW power. Alsulaiman SM [3] reported the natural history and management outcomes of full-thickness macular hole caused by momentary exposure to a high-power handheld blue laser device and concluded that full-thickness macular hole can result from momentary exposure to high-power handheld laser devices.

We present a case of a young man with badly damaged macula due to blue laser exposure in a bar, studied with OCT to evaluate the retinal lesion and mfERG to evaluate functional damage. Out of a curiosity, this young man looked directly at the source of blue laser with his right eye. Later he discovered a central scotoma in his right eye. In our case LED-derived blue laser induced an inner macular hole but not a full-thickness one.

## Case presentation

**Fig. 1:**
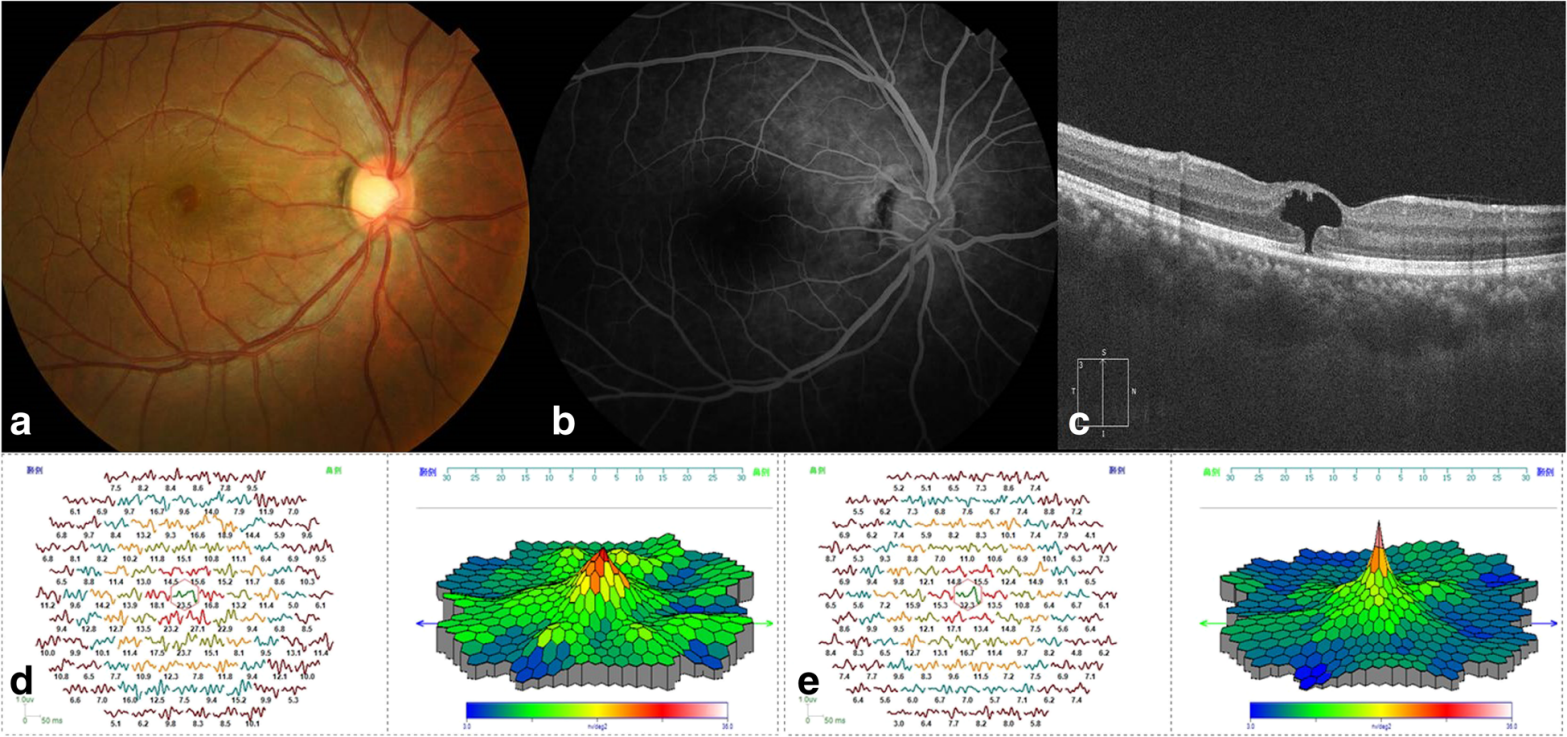
**a** Funduscopy revealed a round red lesion in the macula of the *right* eye. **b** Fluorescein angiography (FA) revealed no leakage. **c** Optical coherence tomography (OCT) revealed a deficiency in the *cente*r of the fovea. **d** MfERG showed a reduction of the peak value in the *right* eye compared to the *left* eye (**e**)

**Fig. 2:**
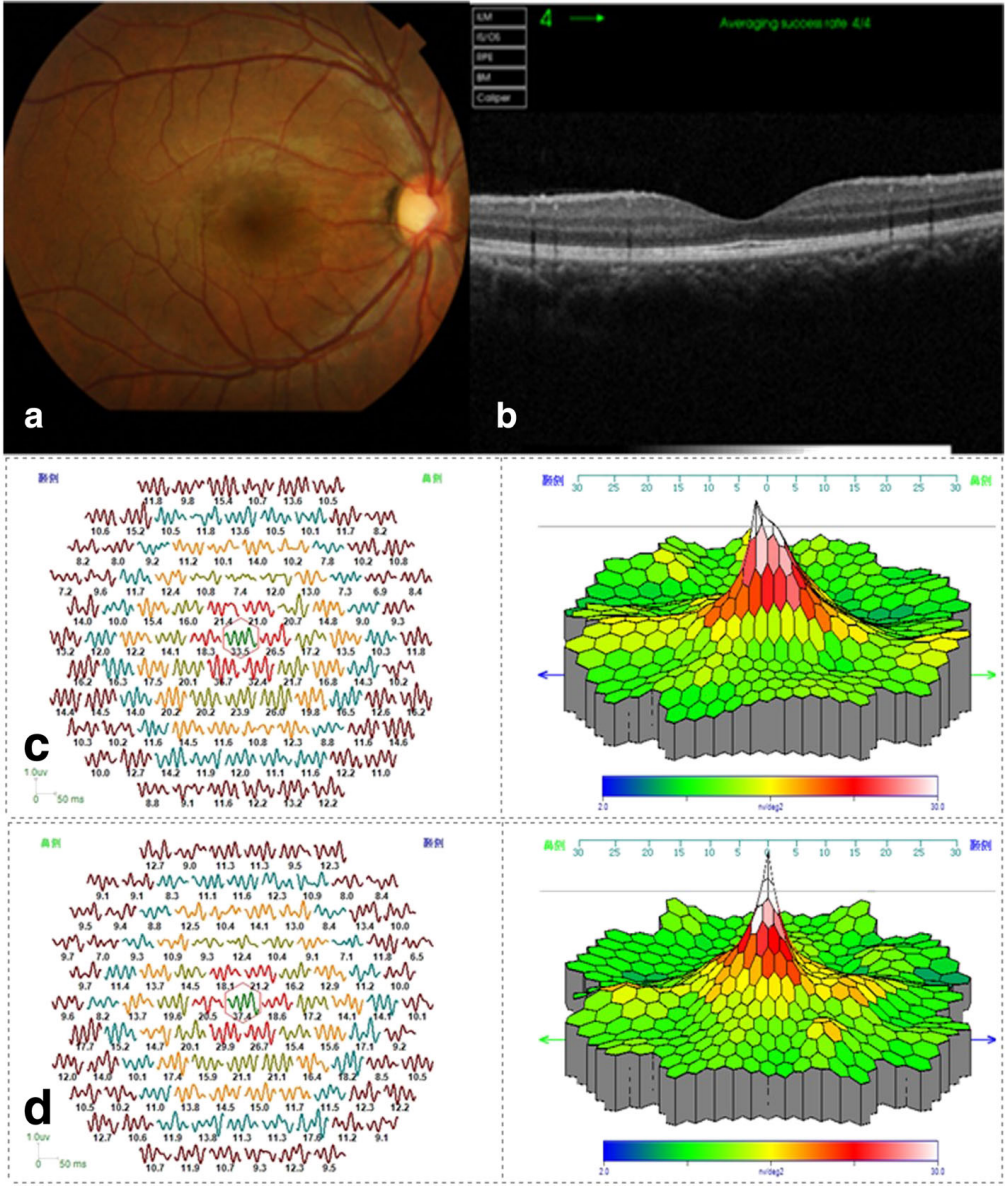
**a** Funduscopy revealed that the round *red* lesion in the macula (Figure 1 **a**) of the *right* eye had disappeared. **b** OCT revealed a hyporeflectivity of the ellipsoid zone (EZ). **c** MfERG still showed a reduction of the peak value in the *right* eye compared to the *left* eye (**d**)

A 29-year-old man complained of central scotoma in the right eye in the early January, 2015. He consulted an ophthalmologist on January 14. Since he did not inform the ophthalmologist of the incident in the bar, the ophthalmologist could not diagnose the disease properly. The young man presented to our hospital on January 18. His visual acuity was 0.5 and eye pressure was 18 mmHg in the right eye. Funduscopy revealed a round red lesion in the macula of the right eye (Fig. 1a). Fluorescein angiography (FA) revealed no leakage (Fig. 1b). OCT revealed a deficiency in the center of the fovea (Fig. 1c). MfERG (GT-2008 V-VI, China) revealed a reduction of the peak value in the right eye compared to the left eye (Fig. 1d). According to the patient’s special experience and clinical manifestation we made the diagnosis of macular damage induced by LED-derived blue **laser**. Lutein, multivitamins, and ginkgo tablet were administered to the patient for four weeks. One month later, `the vision **in** the right eye recovered to 1.0 and the ophthalmoscopic macular morphology returned to normal (Fig. 2a). OCT revealed a hyporeflectivity of the ellipsoid zone (Fig. 2b). MfERG revealed a reduction of the peak value in the right eye compared to the left eye (Fig. 2c).

## Discussion

High-energy visible light has wavelengths in the range of 380 to 530 nm. Blue light (450-495 nm) is high energy visible light [4]. Blue light exposure can induce the mRNA and protein expression of α1D subunit, increasing VEGF and bFGF concentration in retinal pigment epithelial cells [5]. There was a study [6], in which acute blue light damage was applied to eyes of dark Agouti rats over 2 h. Histological analysis confirmed the occurrence of photoreceptor cell death and the development of cellular damage in the outer retina [6]. The peak absorbance of blue light waves has relationship with concentration of xanthophylls in the retina that is, retinal nerve fiber layer (RNFL), inner plexiform layer (IPL), and outer plexiform layer (OPL) hence these are the most probable target tissues [7]. In our case there was a hyporeflectivity from the ellipsoid zone to the RNFL in OCT. This could be attributed to the absence of the tissue because of the photocoagulation effects.

Barkana et al. [8] made a major review discussing the laser induced injuries to the eye extensively. The main laser related factors to determine the eye injuries were the pulse duration and the energy level of the laser beam. Laser radiation can damage the eye by photomechanical, photothermal, or photochemical mechanisms [9]. In our case the LED-derived blue light had a wavelength of 450 nm and 150mw power. The retinal pigment epithelium(RPE) cells absorbed energy from blue light which induced damage to structures above RPE just like the burst of a bomb. So in Fig. 1 the OCT showed a mushroom-shape macular inner hole. Maybe because the energy from blue light was not so strong or the fixation time was not so long that the RNFL escaped from injury. This may cause damage to the macular through photomechanical and photothermal mechanisms.

Mauro Cellini [10] has ever reported a case about arc welding macular injury using OCT and mfERG to evaluate the macular lesion and function. Our case was about blue laser injury and the damage to macular was much more serious than arc welding injury to macular.

In our case even though we observed recovery of visual acuity in the right eye after treatment with antioxidants, OCT showed persistent phototoxic damage in the ellipsoid zone(EZ). In the experimental conditions, protective effects of lutein against blue light-induced retinal damage in rats were significant [11]. Bilberry and lingonberry extracts have protective effect to the retinal photoreceptor cell damage induced by LED blue light in vitro [12]. In this case we can see the protective effect of antioxidants. We will continue evaluating OCT and mfERG for signs of recovery during the follow-up visits.

## Conclusions

In conclusion, we believe that general knowledge about laser injuries to the eye should be realized widely. We also think in cases of macular light damage, the recovery of vision can not demonstrate the recovery of the function of photoreceptors.

## Abbreviations

LED: Light emitting diodes
OCT: Optical coherence tomography
mfERG: multifocal electroretinography
FA: Fluorescein angiography
RNFL: Retinal nerve fiber layer
IPL: Inner plexiform layer
OPL: Outer plexiform layer
EZ: Ellipsoid zone
RPE: Retinal pigment epithelium
